# Integrated NMR and MD structure and dynamics of the stem–loop-II motif (s2m) from the Omicron variant of SARS-CoV-2

**DOI:** 10.1261/rna.080576.125

**Published:** 2025-12

**Authors:** Tobias Matzel, Joseph A. Makowski, Adam H. Kensinger, Andreas Oxenfarth, Maria Wirtz Martin, Jeffrey D. Evanseck, Harald Schwalbe

**Affiliations:** 1 Institute for Organic Chemistry and Chemical Biology, Center for Biomolecular Magnetic Resonance (BMRZ), Goethe-Universität Frankfurt, 60438 Frankfurt, Germany; 2 Department of Chemistry and Biochemistry and Center for Computational Sciences, Duquesne University, Pittsburgh, Pennsylvania 15282, USA

**Keywords:** Covid-19, s2m, RNA, NMR structure, molecular dynamics simulations

## Abstract

The stem–loop-II motif (s2m) is a conserved viral RNA element located in the 3′UTR of different viruses including SARS-CoV-2. High-resolution 3D structural data for s2m are only available for the fundamentally different SCoV-1 version and difficult to access for SARS-CoV-2 due to the highly dynamic nature of the s2m RNA element. With the Omicron variant, a large deletion occurred for s2m, resulting in a relatively short hairpin with an apical pentaloop. We determined the NMR solution structure of s2m_omicron using a variety of torsion-angle sensitive NMR parameters in addition to NOE distance restraints. Surprisingly, relatively high {^1^H},^13^C heteronuclear NOE values, averaged ribose ^3^J_HH_-coupling constants (H1′H2′; H3′H4′), and dipole(H1′-C1′),‐dipole(H6/8-C6/8)-CCRs hinted toward significant dynamics for the small pentaloop making structure calculations solely relying on NMR data insufficient. To address this problem, we performed ten 1 microsecond MD-simulations from the NMR structure bundle as a starting point and applied Bayesian maximum entropy (BME) reweighting to refine the ensemble with the ^3^J-coupling constant data. Our results from the combined methodology provide a detailed view of the conformational dynamics of the Omicron variant of s2m characterized by different stacking patterns, ribose repuckering, and overall heterogeneity of the torsion angles for the loop nucleotides. Strikingly, despite the deletion of the initial nonaloop, as present in the Wuhan and Delta variants of s2m, our combined methodology reveals substantial dynamics and reorganization of a conserved UAC triplet at the tip of the pentaloop, adding physical insight that may be leveraged for the ultimate determination of the still unknown function of the RNA element.

## INTRODUCTION

In 2020, the global Covid-19 pandemic caused by the SARS-coronavirus-2 (SCoV-2) resulted in unprecedented alterations to our daily lives. Up to today, over 770 million cases of Covid-19 have been reported with over 7 million deaths (World Health Organization 2023). While many structures of the SCoV-2 proteome have been unraveled, we still lack structural information of its conserved RNA genome elements. The large viral genome (Wu et al. 2020) consists of ∼30,000 nt. It encodes not only for the viral proteins but also harbors two untranslated regions (UTRs) at its 5′ and 3′ ends. These UTRs contain many different RNA elements that were proposed early in the pandemic (Rangan et al. 2020) and later verified experimentally via NMR spectroscopy and DMS footprinting (Wacker et al. 2020). It has been shown before that the UTRs of coronaviruses are evolutionary conserved and play important roles in the regulation of processes including translation and genome replication (Yang and Leibowitz 2015).

Here, we focus on the stem–loop-II motif (s2m) of the SCoV-2 Omicron variant located within the so-called hypervariable region (HVR) (Goebel et al. 2007) as part of the 3′ UTR of the SCoV-2 genome. s2m is a conserved RNA element that is found close to the 3′ end of many different virus genomes. Initially, it was described in *Astroviridae* (Monceyron et al. 1997) and later also in *Caliciviridae*, *Picornaviridae*, and *Coronaviridae* (Jonassen et al. 1998; Kofstad and Jonassen 2011; Tengs et al. 2013; Tengs and Jonassen 2016). Since these different viruses are not directly related, it was concluded that the element must be transferred horizontally rather than inherited (Tengs et al. 2013). Interestingly, in 2022, a “MixOmicron” hybrid has been reported in SCoV-2 that further elucidates this horizontal transfer. In most subvariants (e.g., 21L/BA.2) of the Omicron lineage, a truncated version of s2m is present (Frye et al. 2023). In contrast, in the 21K/BA.1 variant, a full-length s2m is present that is identical to the s2m found in the original Wuhan version. In this “MixOmicron” genome, the sequence of the 21K/BA.1 variant is combined with a 2500–3000 nucleotide (nt) fragment from 21L/BA.2 restoring the full-length s2m (Colson et al. 2022).

Its evolutionary conservation and widespread occurrence between different species suggest that s2m could play a crucial role in the viral life cycle. It has therefore been successfully targeted using antisense oligonucleotides (ASOs) (Lulla et al. 2021), L-DNA aptamers (Li and Sczepanski 2022), antiviral compounds (Simba-Lahuasi et al. 2022), and low molecular weight compounds identified in a fragment-based NMR screening campaign (Sreeramulu et al. 2021). Following up on this, a close to complete ^13^C chemical shift assignment of the Delta variant of s2m enabled us to identify the primary binding site for these small molecules (Matzel et al. 2024).

Over 25 years after its discovery (Monceyron et al. 1997), the function of s2m has remained elusive. Both the Delta and original Wuhan versions of SCoV-2 s2m have been shown to bind to the host micro-RNA miR-1307-3p that is likely involved in the host immune response through the regulation of cytokines (Imperatore et al. 2022; Cunningham et al. 2023).

In the context of the structure–function paradigm, it is crucial to determine the three-dimensional structures of all viral proteins and viral RNAs. Currently, the number of RNA structures published for SCoV RNA elements is drastically lower than the number of protein structures submitted to the Protein Data Bank (Berman 2000). The substantial lower number of RNA structures is linked to the observation that RNAs can adopt multiple conformations and members of such conformational ensembles undergo substantial dynamics on different time scales as reviewed by others (Mustoe et al. 2014).

Within the Covid19-NMR consortium, we focused on the structure determination of these RNA elements (Wacker et al. 2020; Duchardt-Ferner et al. 2023; Vögele et al. 2023, 2024; Matzel et al. 2024; Mertinkus et al. 2024; Toews et al. 2024; Martin et al. 2025). For the SCoV version of s2m, a crystal structure was published (Robertson et al. 2005), but it has been shown that the base-pairing pattern differs significantly between SCoV and SCoV-2 leading to a different secondary structure (Wacker et al. 2020; Matzel et al. 2024).

Here, we determine the structure and dynamics of s2m_omicron studied by combining NMR with MD. While X-ray crystallography can provide valuable insights into a single stable conformation, NMR ensembles represent a larger fraction of the conformational space of RNAs and allow to explore this plasticity at ambient temperature. NMR structure calculations are predominantly driven by distance restraints from NOEs but dihedral restraints are needed especially for nonhelical regions. For the stem regions, standard A-helical angles can be used as restraints in the structure determination. For smaller loops, the numerous dihedrals within 1 nt are often completely different. J-coupling constants and cross-correlated relaxation rates (CCRs) can be used to calculate angles within these loops to improve the NOE-driven structure calculation (Schwalbe et al. 1993, 1994, 1995; Marino et al. 1996, 1999; Richter et al. 1998; Felli et al. 1999; Rinnenthal et al. 2007). However, especially for dynamic RNAs, it is likely that even with these methods, the obtained structures do not faithfully describe the entire conformational space.

Molecular dynamics (MD) simulations integrate with our NMR analysis by offering atomistic resolution of RNA conformational dynamics, capturing a greater extent of the conformational ensemble in solution than that each can independently achieve. In the past, joint NMR and MD approaches have proven to be especially powerful when studying disordered or dynamic biomolecules (Andrałojć et al. 2016; Chan-Yao-Chong et al. 2019; Bergonzo et al. 2022; Oxenfarth et al. 2023). As RNA structures are often inherently dynamic (Vicens and Kieft 2022), the combined NMR/MD methodology is well suited to describe the RNA structural ensemble. In previous work, the SCoV, SCoV-2, and SCoV-2 Delta versions of s2m have been studied by MD simulations incorporating experimental NOE data for an appropriate starting model (Kensinger et al. 2023; Makowski et al. 2023). However, force field inaccuracies and incomplete sampling can lead to biased or incomplete representations of the true ensemble, particularly for flexible and disordered regions of RNA (Šponer et al. 2018). Consequently, relying solely on unbiased MD simulations risks overlooking low-populated states or mischaracterizing the distribution of conformations. Here, following our previous work, we use a Bayesian/maximum entropy posterior reweighting approach (Oxenfarth et al. 2023). This method addresses the intrinsic underdetermination of the inverse problem posed by experiment, where the number of conformations far exceeds the number of independent experimental measurements, by carefully modifying an ensemble generated by MD to achieve consistency with experimental quantities while preserving the physical realism of the underlying dynamics (Bottaro et al. 2020; Medeiros Selegato et al. 2021; Bernetti and Bussi 2023). Thus, through this strategy, we here present the dynamic structure of s2m_omicron determined via J-coupling, CCR, and NOE-assisted NMR structure determination augmented by MD.

## RESULTS AND DISCUSSION

### The Omicron variant of s2m: secondary structure

**FIGURE 1.**
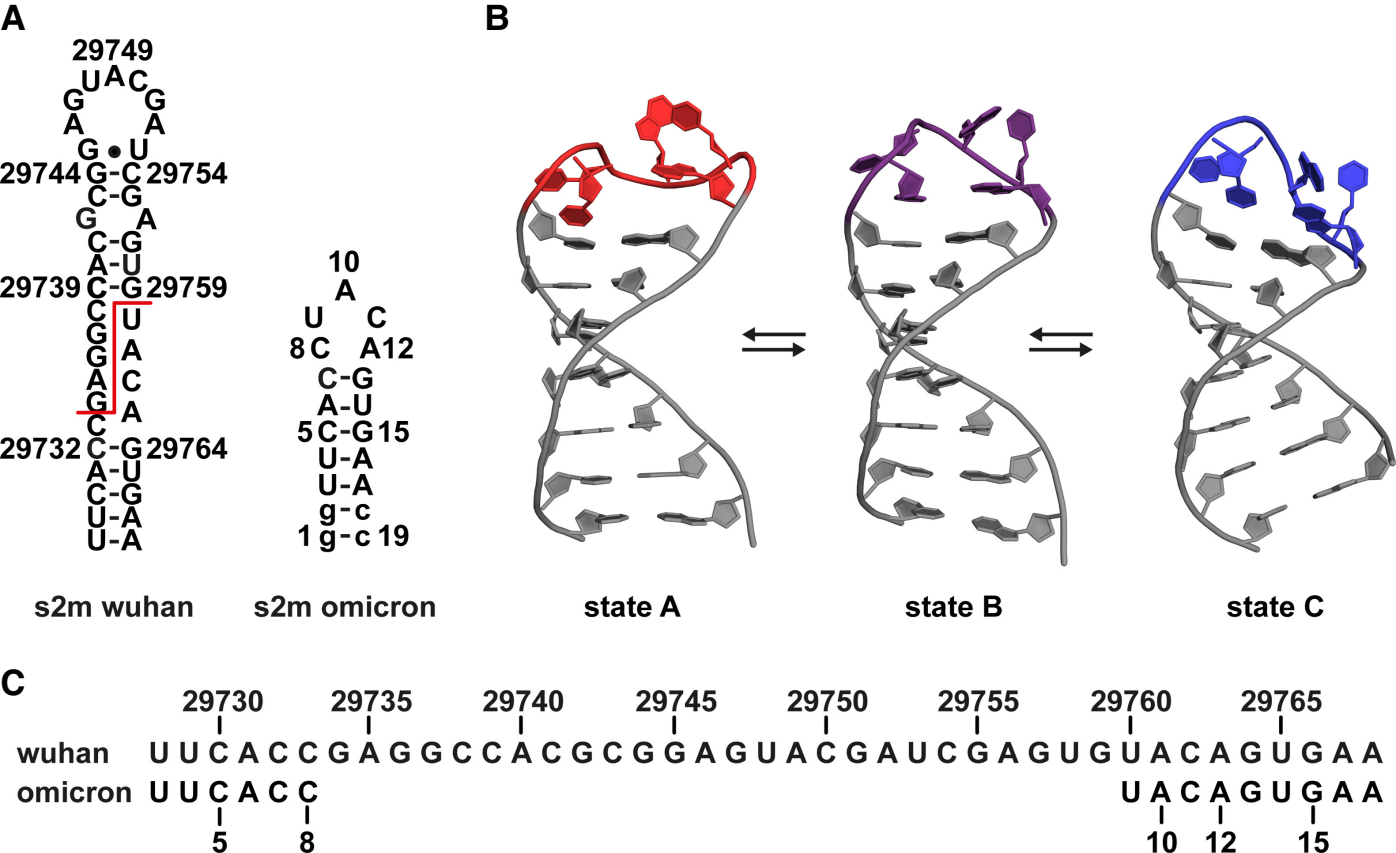
Structural consequences of the s2m_omicron mutation. (*A*) Secondary structure of s2m_Wuhan and s2m_omicron. The position of the deletion is marked with a red line. Artificial nucleotides for T7 in vitro transcription are shown as lowercase letters. (*B*) NMR solution structure of the three main states of s2m_omicron. One representative of each substructure is shown with the loop nucleotides highlighted in color. (*C*) Sequence alignment of s2m_Wuhan and s2m_omicron with the deletion appearing as gap. The nucleotide numbering corresponds to the genomic position of the original Wuhan version -29000.

Mutations to yield the Omicron variant of s2m (s2m_omicron) induced major changes to the secondary structure of the element. While the Delta mutation only induced a single G to U point mutation at position 29742, the most prevalent RNA sequence of the Omicron variant (Fig. 1C) reveals a large deletion of 26 nt from C29734 to G29759 (Frye et al. 2023). This deletion has a major impact on the secondary structure of s2m that now contains only a single stem and a 5 nt terminal loop, from here on referred to as pentaloop (Fig. 1A). The biological relevance of that deletion has remained unclear. It enabled us, however, to analyze the 3D structure of s2m_omicron in detail. NMR structure calculations based on NOE-distance restraints, and both J-coupling constant and cross-correlated relaxation (CCR)-derived dihedral restraints yielded three interconverting main conformational states of s2m_omicron that differ in the nucleotide conformation of the pentaloop (Fig. 1B). In the following section, a detailed characterization of these structures and dynamics is presented. For simplicity, the nucleotides of s2m_omicron will be counted from 1 to 19 from here on, instead of using the genomic numbering.

**FIGURE 2.**
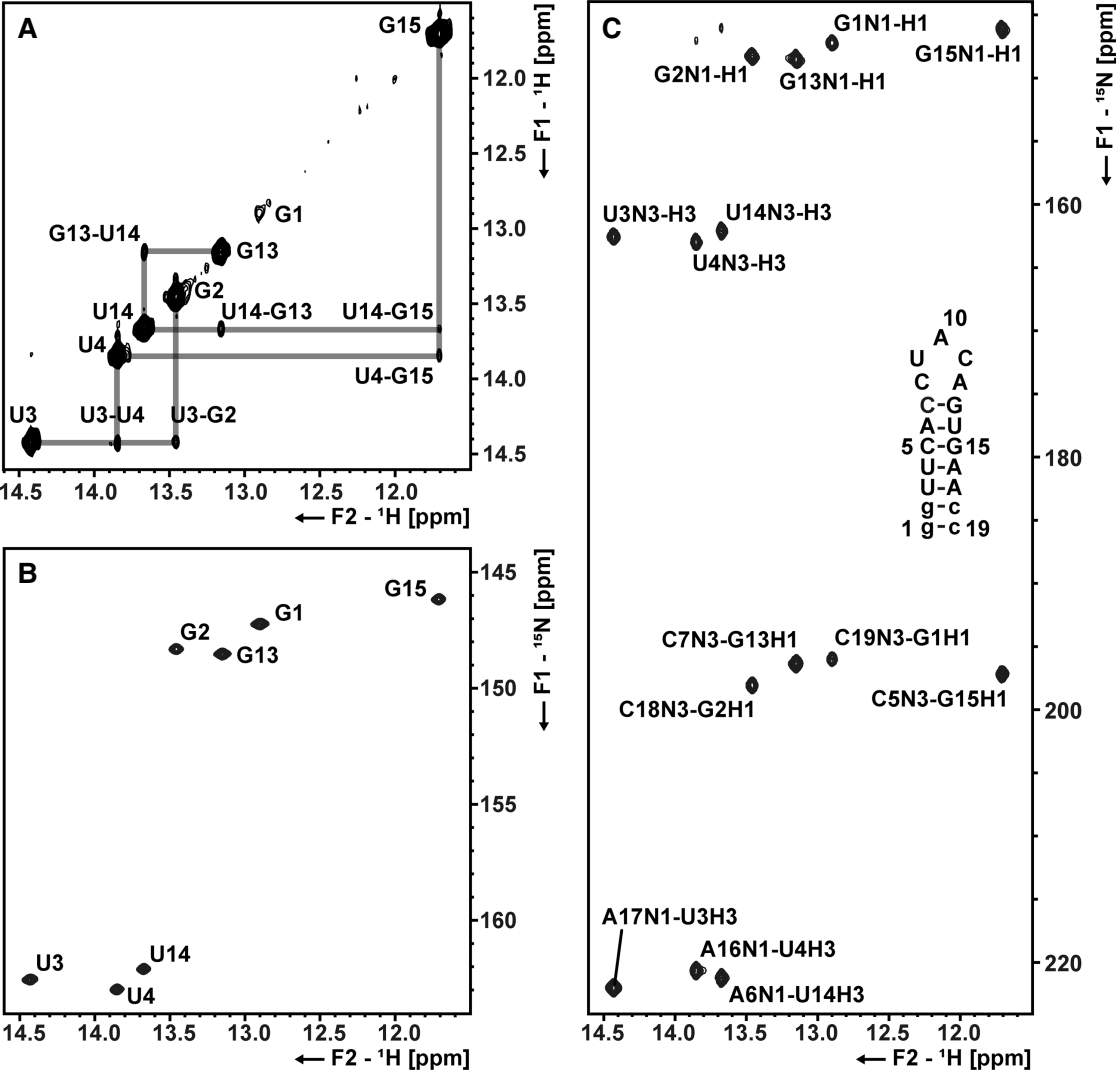
NMR imino proton assignment of s2m_omicron. (*A*) 2D-NOESY of the imino region of s2m. The chemical shift assignments of H1 and H3 imino protons of the G and U nucleotides are annotated. Sequential walks are highlighted as gray lines. (*B*) ^1^H, ^15^n BEST-TROSY spectrum. H1–N1 and H3–N3 correlations are annotated. (*C*) ^1^H, ^15^n HNN-COSY spectrum. Chemical shift assignments are annotated. The secondary structure of s2m_omicron is depicted on the right. Lowercase letters indicate added nucleotides for stability and transcription efficiency. All spectra were measured at 278 K.

To confirm the base-pairing pattern of s2m_omicron, we recorded ^1^H,^1^H-NOESY, ^1^H,^15^N BEST-TROSY, and ^1^H,^15^n HNN-COSY spectra of the imino proton region (Fig. 2). In general, signals of imino protons are only detected if the proton is protected against water exchange, most often due to its involvement in hydrogen bonding. We observed imino proton sequential connectivity along the stem, starting with G2 and ending with G13, matching the assumed secondary structure (Fig. 2A). G and U residues could be distinguished via their characteristic ^1^H and ^15^n chemical shifts in ^1^H,^15^n BEST-TROSY spectra (Fig. 2B). Guanosine H1–N1 chemical shifts are expected between 12.0–13.5 and 145–148 ppm, while uridine H3–N3 chemical shifts are expected between 13–15 and 157–162 ppm, respectively (Nikonowicz et al. 1992; Fürtig et al. 2003). Additionally, we could confirm the standard Watson–Crick (WC) base-pairing for all stem nucleotides in an ^1^H,^15^n HNN-COSY spectrum (Fig. 2C).

**FIGURE 3.**
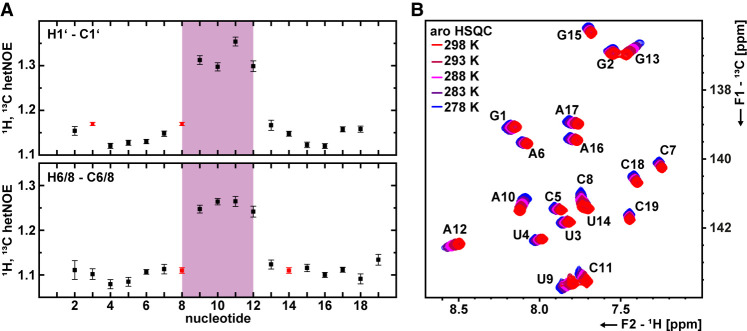
NMR-derived dynamics of s2m_omicron. (*A*) ^1^H, ^13^C heteronuclear NOE of the C1′ ribose (upper panel) and the C8/C6 aromatic resonances (*l*ower panel). The loop of s2m_omicron is highlighted in magenta. Nucleotide C8 overlapped with other resonances and is therefore highlighted in red. (*B*) ^1^H, ^13^C HSQCs of the aromatic H6–C6/H8–C8 resonances from 287 K (blue) to 298 K (red). The resonance assignment is annotated.

After the assignment of the imino protons, we assigned aromatic H6/8-C6/8 and ribose H1′-C1′ resonances using the following 2D and 3D NMR spectra: HCCNH, CPMG-NOESY, HCN, 2D-NOESY, and 3D-NOESY-HSQC. Ribose resonances were assigned using 3D-HCC(H)-TOCSY and fw-HCCH-TOCSY spectra in addition to the 3D-NOESY-HSQC. Assigned ^1^H,^13^C HSQCs of the ribose resonances are shown in Supplemental Figure S1, and the assigned ^1^H,^13^C HSQC of the aromatic H6/8-C6/8 region is shown in Figure 3B. A summary of NMR experiments and parameters is given in Supplemental Table S2, and all assigned resonances are listed in Supplemental Tables S3 and S4. The complete resonance assignment is also provided in the BMRB database (ID: 34991).

### The pentaloop of s2m_omicron is dynamic

The complete assignment of the aromatic H6/8-C6/8 and ribose H1′-C1′ resonances enabled us to analyze the subnanosecond dynamics via {^1^H},^13^C heteronuclear NOE (hetNOE) experiments (Fig. 3A; Kay et al. 1989) in addition to recording the temperature dependence of ^1^H,^13^C chemical shifts of the aromatic resonances (Fig. 3B). H1′-C1′ resonances had a mean hetNOE value of 1.14 for the stem nucleotides. For loop nucleotides, the mean value was 1.32, while the highest value (1.35) was detected for nucleotide C11. In agreement with this, for the aromatic H6/8-C6/8 resonances, we determined a mean hetNOE of 1.10 for the stem and 1.25 for the loop, while the highest hetNOEs (1.26) were detected for nucleotides A10 and C11. Overall, we detected significantly higher hetNOE values for the loop nucleotides U9, A10, C11, and A12. This was well pronounced in case of the aromatic H6/8-C6/8 resonances as well as for the ribose H1′-C1′ resonances. In both experiments, the NMR signals of C8 were overlapping with either U3 (H1′-C1′) or U14 (H6–C6), but as the determined hetNOE of the overlapping peak was small, we concluded that the hetNOE values for C8 also must be relatively small hetNOE value. In agreement with the results of the hetNOE experiments, temperature series of ^1^H,^13^C HSQCs revealed larger chemical shift perturbations (CSPs) for the loop nucleotides compared to the stem nucleotides (Fig. 3B). A CSP plot of the temperature series is given in Supplemental Figure S2. These data indicate structural diversity and dynamics within the loop of s2m_omicron.

### The ribose pucker of the s2m_omicron loop nucleotides is heterogeneous

**FIGURE 4.**
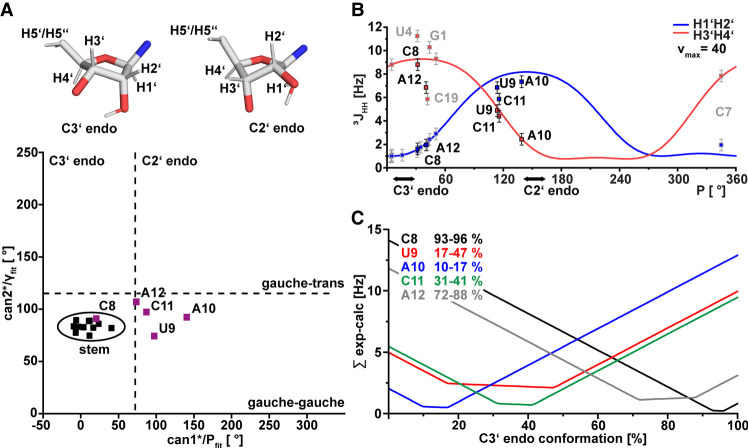
Loop nucleotide ribose conformation of s2m_omicron. (*A*) Canonical coordinates calculated based on the ^13^C chemical shifts of all ribose resonances (see Materials and Methods). Typical regions for C3′ and C2′ endo ribose conformation are separated with dashed lines. The corresponding loop nucleotides are annotated and highlighted in magenta. The ribose conformation of two examples for C3′- and C2′-endo (C5 and A10 from the NMR structure of s2m_omicron) are shown at the top. (*B*) H1′H2′ and H3′H4′ ^3^J-coupling constants determined via the fw-HCC-TOCSY-CCH-ECOSY plotted against the pseudorotation phase based on the Karplus parametrization. H1′H2′ ^3^J-coupling constants are shown in blue, and H3′H4′ couplings are shown in red. Based on the digital resolution of the spectrum, a 0.5 Hz error was estimated. Typical values of the pseudorotation phase for C2′- and C3′-endo are highlighted with bold arrows. Data points corresponding to stem nucleotides are shown in gray contours, while those for loop nucleotides are shown in black contours. (*C*) C3′-endo percentage for each nucleotide determined by globally fitting the ^3^J-coupling constants. The minima of the graphs correspond to populations that best fit the experimental rates.

To investigate the ribose conformation determined by the pseudorotation phase (P), the pseudorotation amplitude ν^max^, and the exocyclic torsion angle (γ), we first calculated canonical coordinates based on the ^13^C assignment of the ribose atoms (Fig. 4A). The calculation of these coordinates is based on an empirical parametrization (Ebrahimi et al. 2001; Cherepanov et al. 2010). Can1* predicts the pseudorotation phase (P) and can2* the exocyclic torsion angle (γ). For all loop nucleotides except C8, the pseudorotation phase was shifted toward C2′ endo conformation. For example, A10 showed a pseudorotation phase of 140.6°. We concluded that A10 predominantly adopts a C2′ endo conformation. U9, C11, and A12 were considered to mainly adopt a C2′ endo conformation; however, this must be interpreted with caution as the predicted pseudorotation phase was not as high as in the case of A10. Strikingly, C8 seemed to adopt a C3′ endo conformation, and it is found in the same region as the stem nucleotides.

Canonical coordinates are parametrized based on the assumption of a single conformation, and the parametrization is based on a limited data set. In a more sophisticated approach to determine the pseudorotation phase of the ribose for all loop nucleotides, we measured ^3^J_HH_-couplings within the ribose via a fw-HCC-TOCSY-CCH-ECOSY experiment. Established pulse sequences (Schwalbe et al. 1995; Glaser et al. 1996) and Karplus parametrization (Haasnoot et al. 1980; Marino et al. 1999) were used to determine P based on the ^3^J-coupling constants. The pseudorotation phase (P) was fitted against two ^3^J-coupling constants (H1′H2′ and H3′H4′) at the same time (Fig. 4B). We determined H1′H2′ and H3′H4′ coupling constants for all the five loop nucleotides (C8, U9, A10, C11, and A12) and most stem nucleotides. Our results indicate that A10 adopts a C2′-endo conformation characterized by a relatively high H1′H2′ ^3^J-coupling constant compared to a small H3′H4′ coupling resulting in a relatively high value of 139° for P. The H1′H2′ ^3^J-coupling constant detected in the experiment was slightly smaller and did not fit perfectly to the theoretical curve. Also, for a pure C2′-endo conformation, pseudorotation phases of ∼162° are expected (Altona and Sundaralingam 1972). In addition to errors potentially caused by the Karplus parametrization, this imperfect fit can be explained by a two-state model with C2′- and C3′-endo conformations present for A10 in the ensemble and their interconversion. In agreement with canonical coordinates, the data suggested that the main conformation of A10 is C2′-endo with lower populated conformational substates in C3′-endo conformation. For U9 and C11, the picture was similar, though the averaging of the ^3^J-coupling constants is even more pronounced than for A10. *P*-values that best fitted the theoretical curves are 114° (U9) and 116° (C11). Both values are in between typical values for C3′-endo (18°) and C2′-endo (162°) with a tendency toward C2′-endo. The coupling constants of C8 and A12 in contrast fit better to the typical A-form helical 3′-endo conformation. Of note also the H3′H4′ ^3^J-coupling constants of some stem nucleotides (U4, G1, and C19) did not fit the expected values. For C19, a smaller H3′H4′ ^3^J-coupling constant can be explained by conformational averaging as it is the last nucleotide of the stem and thereby less rigid. For U4 and G1, the H3′H4′ ^3^J-coupling constant is higher than expected, which can only be explained by intrinsic errors caused by the Karplus parametrization.

To determine the population of C3′- and C2′-endo conformations for each loop nucleotide, we fitted both ^3^J-coupling constants (H1′H2′ and H3′H4′) in a two-state model (see Materials and Methods section) (Fig. 4C). As expected, C8 adopts predominantly (93%–96%) C3′-endo. For U9, we predict a higher uncertainty in the percentage represented by a flat minimum between 17% and 47%; however, the main conformation (53%–83%) of U9 is C2′-endo according to the two-state model. The minimum of A10 appears to be sharper again with 10%–17% C3′-endo. In turn, the ribose of the nucleotide adopts mainly a C2′-endo conformation of 83%–90%. Like U9, C11 adopts mainly a C2′-endo conformation but also (31%–41%) C3′-endo conformation. For A12, the minimum is sharper, yielding a 72%–88% C3′-endo main conformation. To investigate the influence of the chosen reference ^3^J-coupling constants and *P*-values on the determined populations, we performed the same calculations for *P* = 8°, 18°, 28° (C3′-endo) with every possible combination of P = 152°, 162°, 172° for C2′-endo. Only marginal differences in populations were observed (Supplemental Fig. S3).

### Determination of the glycosidic angle χ and the backbone angles β and ε

Another important parameter especially for nucleotides in dynamic regions is the glycosidic torsion angle χ, which characterizes the relative orientation of the ribose and the nucleobase. We determined this angle by measuring the dipole(H1′-C1′)-dipole(H6/8-C6/8)-CCRs Γ^DD,DD^_C1′H1′C6H6_ and Γ^DD,DD^_C1′H1′C8H8_ for purines and pyrimidines, respectively, in a quantitative Γ-HCN. A previously established pulse sequence and parametrization was used to fit the determined rates to theoretical curves (Rinnenthal et al. 2007). The rotational correlation time was determined via HydroNMR (Garcı´a de la Torre et al. 2000) to be 4.7 nsec.

We measured CCRs for C8, U9, and A10. Peaks corresponding to C11 and A12 were not observable in the Γ-HCN spectra. For C8 and A10, we determined CCRs typical for anti-conformation for pyrimidines or purines, respectively (C8: 5.84 Hz, A10: 3.58 Hz). For a pure syn-conformation, much higher rates of up to 35 Hz would be expected. For U9, the determination of χ is ambiguous. We detected a CCR of 9.71 Hz. According to the theoretical curve, this value is in between syn- and anti-conformation. In addition, in our NOESY spectra we detected a cross peak between the H6 proton and the H3′ proton that could only be observed if the nucleotide was in anti-conformation (Supplemental Fig. S4). Taken together, we interpreted the Γ^DD,DD^_C1′H1′C6H6_ value for U9 as ensemble-averaged.

**FIGURE 5.**
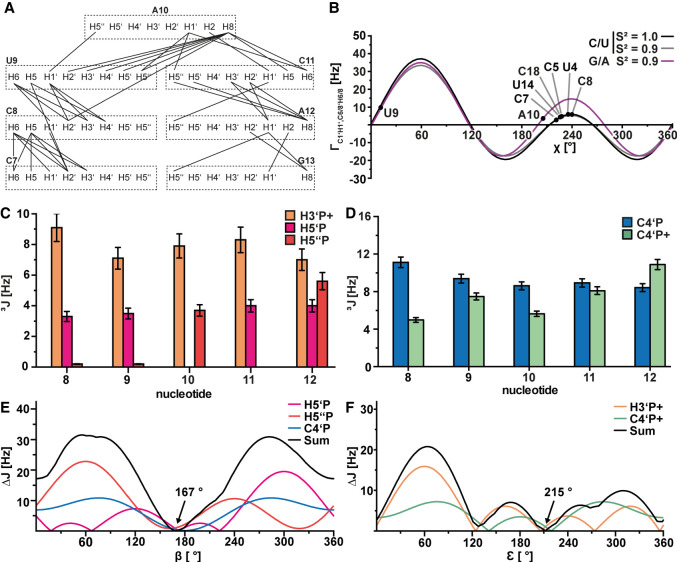
NOE contacts and dihedral angles of the s2m_omicron loop nucleotides. (*A*) Interresidual NOEs within the loop of s2m_omicron. All atoms within 1 nt are shown in a dashed box, and each NOE is represented by a line between two atoms. (*B*) Dipole(H1′,C1′)-dipole(H6/8,C6/8)-CCRs determined via the Γ-HCN experiment plotted against the glycosidic angle (χ) based on the theoretical parametrization. Separate plots are shown for the purines (violet) and the pyrimidines (black, gray). Experimentally determined rates were fitted to the theoretical curves (solid lines) and are shown as dots. Nucleotide names are annotated. (*C*) ^3^J H-P couplings determined via the PFIDS experiment. $H3_{i}^{\prime }-P_{i+1}$ couplings are shown in orange, $H5_{i}^{\prime }-P_{i}$ couplings are shown in magenta, and $H5_{i}^{\prime \prime }-P_{i}$ couplings are shown in red. A 10% error was estimated. (*D*) ^3^J C-P couplings determined via the qHCP experiment. $C4_{i}^{\prime }-P_{i}$ couplings are shown in blue, and $C4_{i}^{\prime }-P_{i+1}$ couplings are shown in green. A 5% error was estimated. (*E*) Determination of the backbone angle β for nucleotide U9 based on $H5_{i}^{\prime }-P_{i}$ couplings (magenta), $H5_{i}^{\prime \prime }-P_{i}$ couplings (red), and $C4_{i}^{\prime }-P_{i}$ couplings (blue). (*F*) Determination of the backbone angle ε for nucleotide U9 based on $H3_{i}^{\prime }-P_{i+1}$ couplings (orange) and $C4_{i}^{\prime }-P_{i+1}$ couplings (green). Each curve corresponds to the experimentally determined coupling minus the theoretical value of the Karplus parametrization plotted against the angle. The sum of these curves is shown in black. The minimum is annotated and gives the angle that best fits all used couplings.

The determination of the phosphodiester backbone angles β and ε was carried out by measuring different H-P (Fig. 5C) and C-P couplings (Fig. 5D) and subsequent fitting to Karplus parametrization (Lankhorst et al. 1984). The C-P ^3^J-coupling constants $C4_{i}^{\prime }i-P_{i}$ and $C4_{i}^{\prime }-P_{i+1}$ were determined via a quantitative HCP (Richter et al. 1998), and the H-P J-coupling constants $H3_{i}^{\prime }-P_{i+1}$, $H5_{i}^{\prime }-P_{i}$ and $H5_{i}^{\prime \prime }-P_{i}$ were determined via a PFIDS experiment (Schwalbe et al. 1993). The determination β was carried out by simultaneously fitting the measured $H5_{i}^{\prime }-P_{i}$, $H5_{i}^{\prime \prime }-P_{i}$, and $C4_{i}^{\prime }-P_{i}$ rates to the theoretical curves. The minimum in the sum plot then yields the dihedral angle that fits best to all couplings. In the same manner, ε was determined via the $H{3^{\prime }}_{i}-P_{i+1}$ and $C4_{i}^{\prime }-P_{i+1}$ rates. As an example, the parametrization for nucleotide U9 is shown (Fig. 5E,F). We determined β and ε for all loop nucleotides (Supplemental Table S5). The thus determined angles were included in the structure calculation.

### NMR solution structure of s2m_omicron

For the structure calculation of s2m_omicron, we implemented additional restraints beyond common NOE distances. Specifically, we implemented dihedral angle restraints that were determined via different J-coupling constant or CCR-based NMR methods (Figs. 4, 5). A list of all experimentally determined dihedral restraints for the loop is given in Supplemental Table S5, and all standard dihedral restraints for the stem are given in Supplemental Table S6. To prevent overrestraining of the loop nucleotides, intervals were used as dihedral restraints rather than exact angles. In total, 254 NOE distance restraints (13.4 per nucleotide) were incorporated in the structure calculation (Fig. 5A). For the five loop nucleotides, 88 NOEs restraints were used (on average 17.6 per nt), with 41 interresidual NOEs (included are NOEs from C8 to C7 and from A12 to G13). As emphasized by the results shown above, we restrained C8 and A12 in C3′-endo conformation and A10 in C2′-endo conformation. For our structure calculation, we also included restraints for C2′-endo conformation of U9 and C11. As evidenced by the CCR data, we restrained C8 and A10 in anti-conformation and applied no restraints for U9. Since we detect a NOE cross peak between H6 and H3′ for U9, there must be anti-conformation present to a certain extent. At the same time, the CCR for U9 is much higher than expected for only anti-conformation indicating a population in syn-conformation.

**FIGURE 6.**
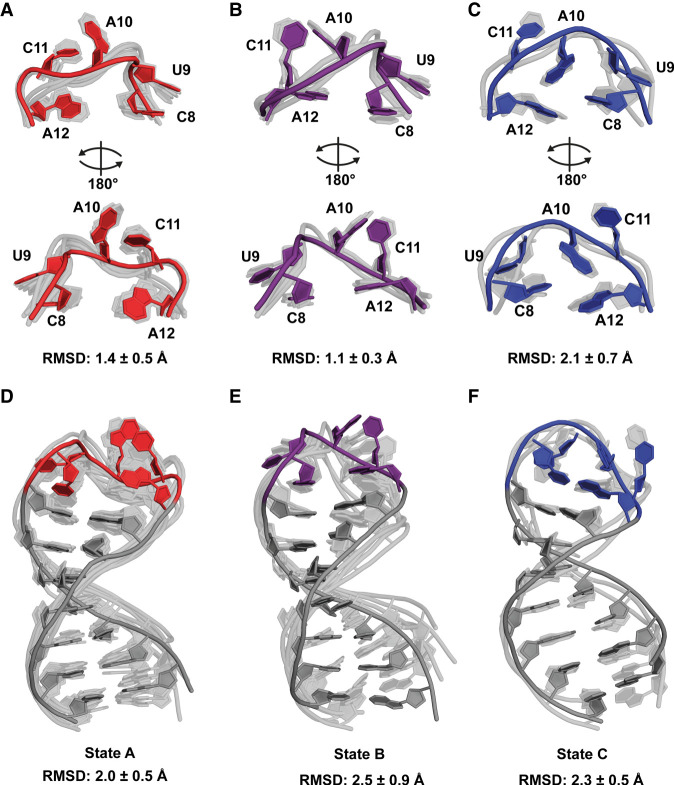
Three-state NMR solution structure of s2m_omicron. Out of the total 20 structures, nine structures are assigned to state A, five structures to state B, and three structures to state C. The bundles are shown transparent, and one representative is shown in gray with the loop nucleotides in color and annotated. (A + B + C) Aligned loop nucleotide of state A (*A*), state B (*B*), and state C (*C*) with pairwise mean RMSDs of 1.4 ± 0.5, 1.1 ± 0.3, and 2.3 ± 0.5 Å, respectively. (D + E + F) Aligned full NMR structures of state A (*D*), state B (*E*), and state C (*F*) with pairwise mean RMSDs of 2.0 ± 0.5, 2.5 ± 0.9, and 2.3 ± 0.5 Å, respectively. The structure bundle is accessible via the PDB (code: 9QZJ).

The resulting structure bundle after the structure calculation with ARIA consisted of 20 structures and could be divided into three main states that were characterized by differences in the relative orientation of A10 and C11 (Fig. 6). Nine out of the 20 structures were assigned to state A, five to state B, and three to state C. The remaining three structures could not be assigned to any of the other states and are seen as “exotic states.” As measure of variance (not of quality) within each state, we calculated the pairwise mean RMSDs using MOLMOL (Koradi et al. 1996). Within the loop nucleotides, state A has an RMSD of 1.4 ± 0.5 Å, state B has an RMSD of 1.1 ± 0.3 Å, and state C has an RMSD of 2.1 ± 0.7 Å. For the entire RNA, states A, B, and C have RMSDs of 2.0 ± 0.5, 2.5 ± 0.9, and 2.3 ± 0.5 Å, respectively. The RMSD of the full bundle increases after adding stem nucleotides because the loop can no longer independently be aligned in this case. Aligning only the loop or only the stem independently of the other section therefore results in a decreased RMSD compared to aligning the full RNA. We do not expect larger rearrangements in the stem. In all substructures, the loop opening nucleotides C8 and A12 show very similar arrangements characterized by base stacking with the stem nucleotides as expected in a standard A-form-helix. In contrast, nucleotide U9 is flipped out and points away from the rest of the loop in all substructures. The main difference between all structures is based on the relative orientation of A10, C11 and A12. State A is characterized by a base stacking of A10 and C11, while A10 is located above C11. In states B and C, A10 is stacking with A12 and located on the same level or below C11, while the latter one is flipped perpendicular to A10 and A12 to make room for their interaction. The difference between states B and C is that state B seems to represent an intermediate between the states A and C where A10 and C11 are almost on the same vertical plane. In contrast, in state C, A10 is clearly located below C11. We interpret the three states as the main conformations of s2m_omicron. However, there are possibly several additional states that are not represented in the structure bundle. To expand the view on the conformational space of s2m_omicron and to investigate how different states interconvert, we carried out molecular dynamics (MD) simulations starting from the NMR structures.

### Molecular dynamics simulations extend the view on the dynamics of s2m_omicron

While NMR provided valuable insight into the overall secondary structure and local conformational features, averaging indicated that dynamics were obscured and suggest the presence of a heterogeneous structural ensemble that cannot be fully resolved through experimental observables incorporated into NMR structure calculation protocols alone. Thus, molecular dynamics (MD) simulations were used as a means of generating a structural ensemble to bring clarity to the observed experimental averages and ultimately describe the structure and dynamics of s2m in solution.

**FIGURE 7.**
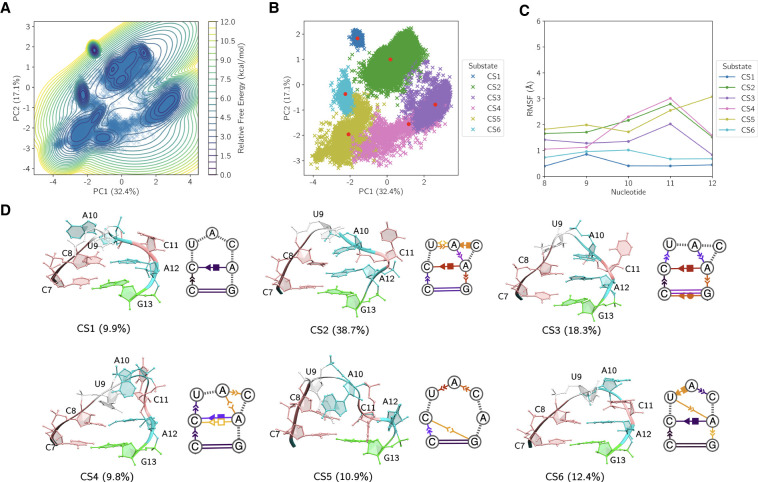
(*A*) Principal component projection of the MD trajectories, with estimated free energy contours revealing a structured conformational landscape containing multiple low-energy basins. (*B*) Spectral clustering identifies six conformational substates (CSs), with representative centroids indicated as red points. (*C*) Root mean square fluctuation of the terminal loop nucleotides within each CS relative to its centroid, highlighting substate-specific flexibility. (*D*) Three-dimensional centroid structures, substate populations, and secondary structures annotated using the Leontis–Westhof nomenclature within each CS, where base-pairing and nucleobase stacking propensities are visualized via color intensity (darker tones indicate higher probability across the cluster ensemble).

The aggregated MD data were projected into a low-dimensional subspace using principal component analysis (PCA), capturing the dominant motions across the ensemble. The dynamic dependence on starting structure and the extent of individual simulation sampling is depicted in Supplemental Figure S5, showing our MD trajectories visit conformations distinct from the solution NMR starting structures, equilibrate between multiple distinct states, and multiple simulations tend to visit the same states independently. The first two principal components (PC1 and PC2), accounting for ∼50% of the variance (32.4% and 17.1%, respectively), reveal a structured free energy landscape with multiple distinct basins (Fig. 7A). A free energy estimation using the projected density highlights the presence of several metastable conformational substates (CSs). Spectral clustering was applied to partition the structures defining this landscape into six CSs (Fig. 7B). Conformational variability in the ensemble was assessed through analysis of representative centroids (red points) from each substate. To probe the internal dynamics of each substate, we computed the root mean square fluctuation (RMSF) of each nucleotide relative to its centroid structure (Fig. 7C). This analysis reveals only small substate-specific differences in pentaloop flexibility, where all nucleotides remain on average within 3 Å of their centroid reference structures in each CS, suggesting that differences between the six centroids are sufficient for describing large structural changes in the simulation data. Centroid structures and secondary structures using the Leontis–Westhof nomenclature for each CS are shown in Figure 7D (Leontis and Westhof 2001).

The six substates encompass a range of distinct conformational motifs, including closed, partially open, and unstacked loop geometries. Based on the estimated free energy from PCA and corresponding intra-CS RMSF, CS1 (9.9%) is a kinetically trapped and structurally homogenous state sampled within the MD data. The dynamic secondary structure reveals that this cannot be rationalized by standard nucleobase stacking or noncanonical base-pairing. Closer inspection of the 3D structure and hydrogen bonding within CS1 shows persistent hydrogen bonds (Supplemental Fig. S6; Supplemental Table S9); specifically, beyond noncanonical base-pairing between A12 H6 and C8 O2 (88.3% population) similar to other CSs, the A10 phosphodiester plays an important role in restraining U9 and C11 by bending into the loop and forming hydrogen bonds with U9 HO2′ (91.0% population) and C11 H4 (82.3% population), limiting each nucleotide's dynamics within CS1. Furthermore, the free rotation of A10 about χ remains limited throughout CS1 due to its proximity to the phosphodiester of U9. In contrast, CS2 is the most populated substate (38.7%) with dynamics well-described by base stacking and noncanonical base pairs. Similar to CS1 and most other CSs, CS2 contains a prevalent *cis* Hoogsteen/sugar-edge base pair between A12 and C8 and an A10/A12 nucleobase stack. The dynamic secondary structure also reveals U9 torsion about χ between syn and anti while stacking with A10, resulting in an organized loop structure characterized by a stack between U9, A10, A12, and G13 for much of CS2. While C11 is swung out and solvent exposed, reflected in its elevated RMSF, it occasionally base-pairs with A10. Next, while CS3 (18.3%) contains many of the secondary structure features mentioned previously, it differs from CS2 insofar as it contains two distinct C7/U8/U9 and A10/A12/G13 nucleobase stacks, with U9 flipped backward relative to its orientation in CS2. CS4 exhibits dynamics in the A12 nucleobase rotating about its glycosidic bond, where the A12-C8 Hoogsteen/sugar-edge base pair was observed to isomerize from *cis* to *trans* due to A12 sampling the syn-conformation. The nucleobase of A10 is also dynamic, stacking with the nucleobase of A12 in a different torsional state for a smaller proportion of the substate. CS5 is characterized by A12's nucleobase flipping out, disrupting its stack with the nucleobase of A10 and leading to an open, disordered loop structure with only fleeting nucleobase stacking. Likewise, CS6 captures a conformation of the loop wherein the A10/A12 nucleobase stack is not present, replaced by a fleeting U9/A12 nucleobase stack and a more highly populated A10/C11 nucleobase stack due to both nucleotides flipping to the back of the ring.

**FIGURE 8.**
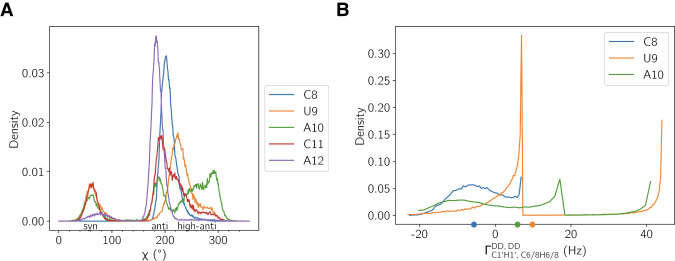
(*A*) Distribution of the χ dihedral angle sampled over MD trajectories for each pentaloop nucleotide. (*B*) Cross-correlated relaxation (CCR) rate calculated from MD, where mean values are given by dots (C8 = −4.4 Hz; U9 = 10.1 Hz; A10 = 6.3 Hz).

To assess the degree to which our MD ensemble captures experimentally measurable dynamic signatures, we compared computed χ dihedral distributions and CCRs for the pentaloop residues against available NMR data (Fig. 8). In line with the experimental data, C8 exclusively adopts an anti-conformation with a mean χ angle of 206.9°, consistent with both its role as a relatively stable nucleotide with a lack of significant deviation in its local environment across substates. However, the computed CCR value of −4.4 Hz for C8 underestimates the experimentally observed 5.8 Hz, a discrepancy which arises from insufficient sampling of syn substates on the timescale of 10 µsec of MD data. This underestimation suggests a kinetic barrier to alternative conformers that could impact CCRs but are not resolved in the trajectory due to insufficient transitions or poor ergodicity in this coordinate.

In contrast, U9 displays a bimodal distribution in χ, with significant sampling of both syn- and anti-conformations with a mean torsion of 226.0°. The computed CCR rate of 10.1 Hz is in strong agreement with the experimental value of 9.7 Hz, which had previously been interpreted as indicative of a dynamic average between conformational states. The ability of MD to reproduce this intermediate rate provides further evidence that U9 undergoes χ torsion fluctuations, a feature that could not be satisfactorily captured by restrained structure calculations or static ensemble models. Furthermore, the presence of an H6–H3′ NOE cross-peak in experiment, which implies transient adoption of an anti-conformation, is consistent with the antipopulation resolved in our simulations.

A10 exhibits more diffuse sampling with populations in syn, anti, and high-anti regions. The MD-derived mean χ of 248.9° and the corresponding CCR of 6.3 Hz slightly deviate from the experimental value of 3.6 Hz. While this discrepancy is modest, it may reflect oversampling of higher-anti conformations or interactions not adequately sampled in this ensemble. Nevertheless, the presence of a multimodal χ distribution aligns qualitatively with the expectation of conformational flexibility at this position from hetNOE experiments.

Overall, these data substantiate the view that U9 is not adopting a single χ conformation but exists in a dynamic distribution of conformations. The analysis of χ conformation for additional nucleobases was not possible since the computed CCRs deviated from experiment, suggesting that even longer MD trajectories are necessary to obtain a distribution of states consistent with these experimental measurements.

Despite the overall fidelity of our MD ensemble relative to experimentally observed structural and dynamical properties, a more nuanced discrepancy emerges when considering ^3^J scalar couplings in the pentaloop region. While the stem residues exhibit strong agreement between calculated and experimental ^3^J-coupling constants (Supplemental Fig. S7A), the loop nucleotides show deviations with NMR data. These discrepancies suggest that while the MD ensemble adequately captures the global fold and long-lived substates, it likely misrepresents the relative populations of local ribose conformations within the loop. To address this, we used BME reweighting to refine the simulation ensemble by incorporating experimental ^3^J scalar couplings as additional constraints. The BME approach offers a principled means to correct ensemble bias by minimally perturbing the original MD ensemble, subject to the condition of improved agreement with observables. In this framework, each frame from the simulation is assigned a new statistical weight, optimized to reduce the mismatch with experimental ^3^J values while preserving the underlying diversity of the sampled conformational space.

As shown in Supplemental Figure S7A, this reweighting dramatically improves the match between calculated and experimental couplings in the pentaloop, with negligible impact on the already satisfactory agreement observed for the stem. Importantly, this enhancement does not come at the cost of excessive reduction in ensemble diversity. In Supplemental Figure S7B, we observe a relatively high proportion of effective frames retained post-reweighting, even with a hyperparameter choice favoring substantial correction (θ = 3). This outcome indicates that the original MD ensemble contains the necessary conformational heterogeneity to reconcile with experimental ^3^J-coupling constants, and that the misalignment was primarily due to inaccurate population weights rather than absence of relevant ribose conformations.

**FIGURE 9.**
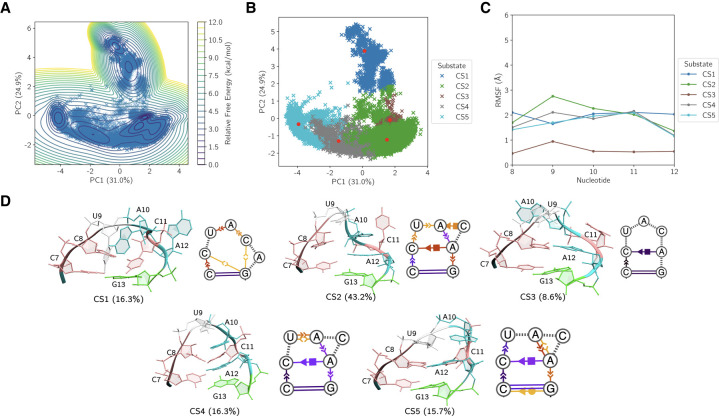
(*A*) Principal component projection of an optimized ensemble based on ^3^J-couplings, with estimated free energy contours revealing a structured conformational landscape containing multiple low-energy basins. (*B*) *K*-means clustering identifies five dominant conformational substates (CSs), with representative centroids indicated as red points. (*C*) Root mean square fluctuations of the terminal loop nucleotides within each CS relative to its centroid. (*D*) Three-dimensional centroid structures, substate populations, and dynamic secondary structures within each CS, where base-pairing and nucleobase stacking within each CS are visualized via color intensity (darker tones indicate higher probability across the cluster ensemble).

The conformational landscape of the pentaloop following BME optimization reveals a reorganized but fundamentally consistent substate architecture, wherein key features of the unweighted MD ensemble are preserved, and subtle corrections are introduced to improve consistency with experimental scalar couplings. PCA applied to the reweighted ensemble (Fig. 9A) reveals an energy landscape consisting of fewer distinct basins and different topology, reflective of a change in the most prominent dynamics suggested by the principal components. Clustering identifies five dominant substates (Fig. 9B), each of which exhibits clustered geometries with low intrasubstate fluctuations (Fig. 9C), reaffirming that the centroid structures (Fig. 9D) are representative of the structures within each CS and that the reweighted distribution retains its conformational rigidity within substates.

The distributions of sampled secondary structure populations derived from MD trajectories and BME are presented in Supplemental Figure S8, where structures were categorized according to their resemblance to three experimentally motivated states: “State A,” “State B/C,” and “Exotic,” as defined previously. In the MD ensemble, CS2, CS3, and CS4 (total 66.8%) adopt conformations assigned to “State B/C.” A smaller fraction (10.9%) corresponds to “State A,” derived solely from CS5. The remaining 22.3% represents “Exotic” structures, attributable to CS1 and CS6, which exhibit structural features inconsistent with the reference states and highlight the conformational diversity accessible to the force field in the absence of experimental restraint.

Reweighting the ensemble with BME, using experimental ^3^J scalar couplings as constraints, shifts these populations in a manner reflecting the direct influence of ribose pseudorotation on local structure. The BME procedure, by construction, optimizes ensemble weights to match experimental scalar couplings, which are themselves sensitive reporters of ribose ring puckering and local backbone geometry. This targeted correction is manifest in the removal of MD CS6 from the BME ensemble; its ribose conformations evidently yield coupling constants incompatible with the measured values, resulting in its suppression post-reweighting. As a result, the “Exotic” population contracts from 22.3% in MD to 8.6% in the BME-refined ensemble, which is based solely on BME CS3, which corresponds structurally to MD CS1. This cluster persists with minimal reduction in prevalence (from 9.9% to 8.6%).

The canonical states also experience subtle adjustments under BME refinement. The “State A” population grows modestly to 16.3% due to BME CS1, which structurally resembles MD CS5 but exhibits enhanced base stacking interactions that likely shift its local geometries into closer agreement with the scalar coupling constraints. Meanwhile, the “State B/C” fraction expands to 75.1%, comprised of BME CS2, CS4, and CS5. Notably, MD CS2 and BME CS2 correspond to the same state and remain the single most populated cluster before and after reweighting.

These results demonstrate that the unbiased MD ensemble captures the dominant solution-phase conformational states with reasonable fidelity but also generates populations of ribose geometries that fail to reproduce experimentally measured couplings, particularly CS6. These inconsistencies were improved in the BME ensemble by reducing the weighting of incompatible substates and redistributing their population toward structures with ribose pseudorotation phases consistent with the scalar coupling data. The persistence of “Exotic” fractions even after reweighting suggests that some structural heterogeneity remains consistent with ribose dynamics.

**FIGURE 10.**
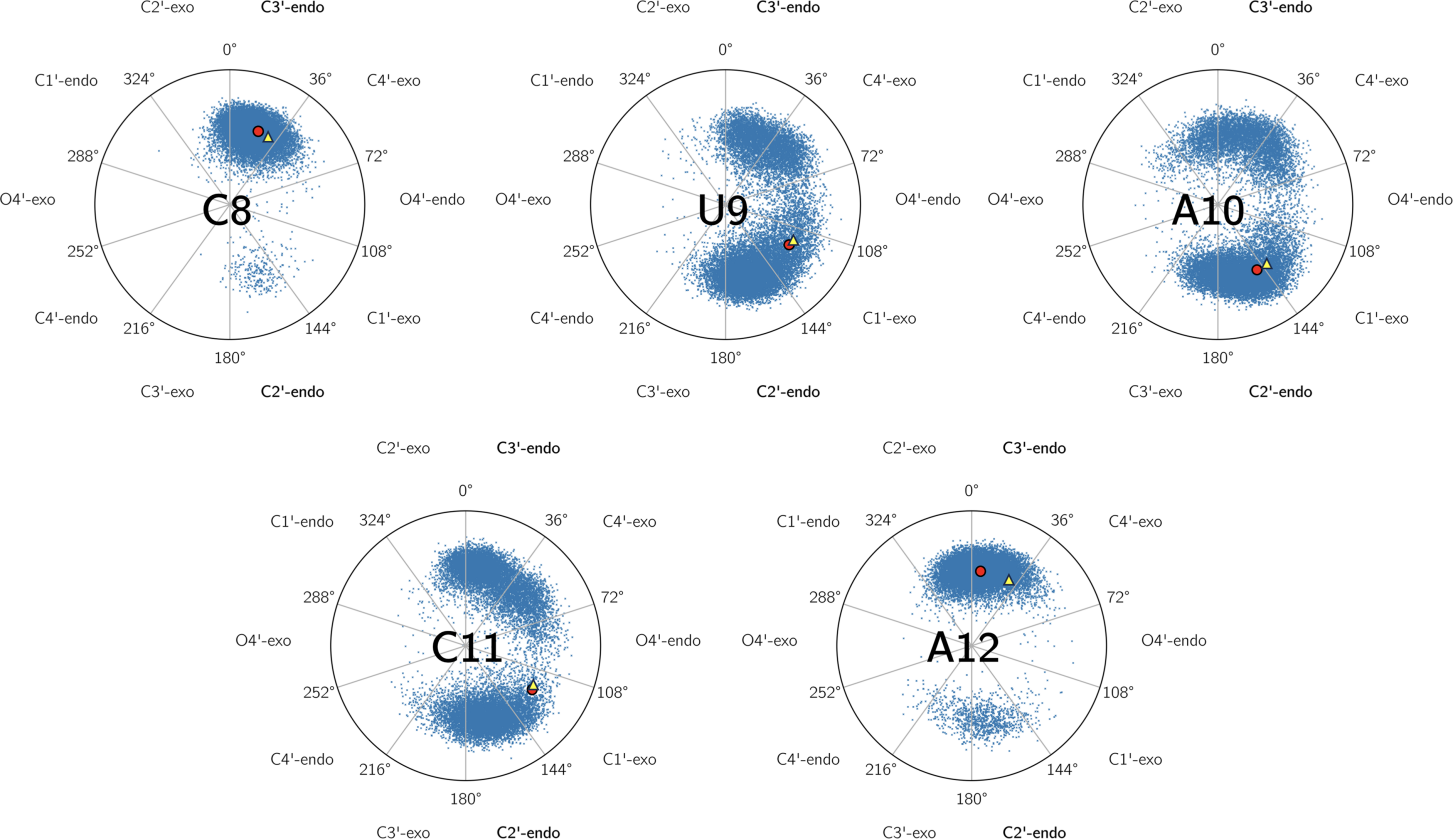
Pseudorotation angles and amplitudes of the ^3^J reweighted ensemble of pentaloop nucleotides. The computed mean is given by a red dot, whereas the pseudorotation phase from H1′H2′ and H3′H4′ ^3^J-couplings is given by yellow triangles.

The reweighted ensemble derived from Bayesian maximum entropy optimization guided by H1′H2′ and H3′H4′ ^3^J-couplings displays ribose pseudorotation angles with improved agreement with experimentally inferred conformations (Fig. 10). The mean pseudorotation phases and amplitudes computed across the reweighted ensemble cluster around experimentally determined values based on theoretical curve fitting, confirming that the ensemble correction not only improved scalar couplings but also provided a plausible distribution of pseudorotation phases for interpretation with experiment. While the distribution of pseudorotation phases from the MD ensemble achieved similar agreement between the computed average and experimental values for C8, U9, and A12, reweighting markedly improved the distributions of A10 and C11 (Supplemental Fig. S9).

As expected, C8 remains dominant in the C3′-endo conformation, consistent with both canonical coordinate predictions and the elevated H3′H4′ ^3^J-coupling constant observed experimentally. The reweighted pseudorotation distribution remains tightly localized near 18°, indicating a robust and well-sampled C3′-endo population that reflects its stability due to persistent noncanonical base-pairing with A12, where a similar result shows a mean pseudorotation angle closely aligned with C3′-endo. A10, in contrast, shifts decisively toward a C2′-endo geometry in the reweighted distribution, in line with the high pseudorotation phase (∼140°) estimated from NMR, and consistent with a small H3′H4′ ^3^J-coupling constant and large H1′H2′ ^3^J-couping. The mean phase value in the reweighted ensemble agrees, and the distribution reveals minor occupancy of C3′-endo, consistent with the mixed conformational signature implied by imperfect experimental fits. Notably, this modest conformational diversity is retained despite BME optimization, suggesting that reweighting promotes the correct ensemble mean without artificially constraining flexibility. For U9 and C11, the reweighted distributions reveal broad pseudorotation phase populations centered between 100° and 120°, reflecting a true conformational averaging between C2′- and C3′-endo geometries. The experimental data similarly indicate that these nucleotides exist in mixed sugar pucker states, with flatter fitting curves and increased uncertainty in estimated C3′-endo populations. The ensemble distribution provides physically reasonable sampling in alignment with the two-state models from experiment, averaging to intermediate pseudorotation values due to significant populations of both C2′- and C3′-endo ribose conformations. The reweighted ensemble preserves sugar pucker heterogeneity in nucleotides where the experimental data demand it (U9, C11), and sharpens conformational distributions only where the experimental data support a clear dominant state (C8, A10, A12). These results underscore the capacity of BME to extract experimentally consistent microstate populations without compromising the structural diversity of the underlying simulation.

Beyond the ^3^J-coupling constants targeted for reweighting, we find small differences in the χ torsion distributions and resultant CCRs (Supplemental Fig. S10). Due to a lack of sufficient sampling of the syn-conformation, the computed C8 CCR from the reweighted ensemble (−3.6 Hz) remains an underestimate relative to experiment. On the other hand, U9 samples the anti-conformation less frequently in the reweighted ensemble, resulting in an overestimate of the CCR (16.2 Hz), but A10 is brought into closer alignment with experiment (3.0 Hz) due to greater sampling of the high anti-conformation at the expense of anti. These findings suggest that the C8 conformational landscape remains “undercorrected” due to its limited sampling of other χ states in the underlying MD data; U9 was “overcorrected,” given that both its pseudorotation and χ distributions were already in agreement with experiment prior to reweighting. The successful correction of the pseudorotation phases of A10, which were initially in error with experiment, led to a more accurate χ torsional profile as well. These observations underscore the importance of avoiding overfitting, retaining structural diversity when using posterior ensemble reweighting procedures, and drawing individual conclusions carefully from possibly multiple ensembles (MD-derived and BME optimized) to achieve the most realistic description of structure and dynamics.

**FIGURE 11.**
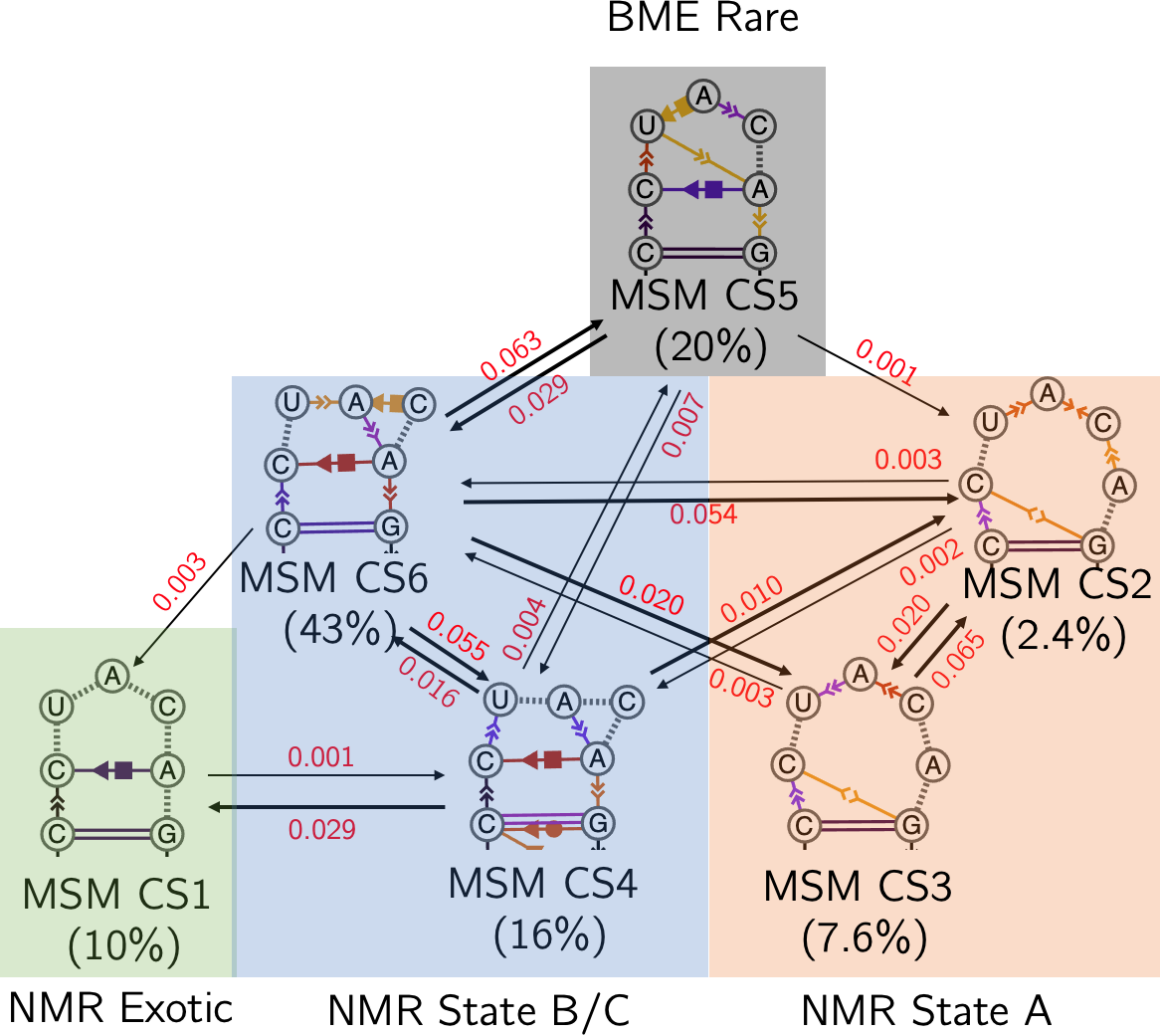
Markov state model depicting transitions between six CSs derived from MD simulations, with each node represented by a dynamic secondary structure schematic incorporating both base-pairing and nucleobase stacking. Directed edges represent observed transitions, with arrow thickness and edge labels indicating the respective transition probabilities at a lag time of 30 nsec. Percentages below each MSM CS represent the proportion of frames from the MD-derived ensemble falling within the MSM macrostate. Self-transitions are not shown.

To quantify the kinetic interconversion between states sampled through MD, we constructed a Markov state model (MSM) based on the transitions between states sampled within each of the 10 MD trajectories. Using a lag time of 30 nsec, validated by convergence of implied timescales (Supplemental Fig. S11) and the Chapman–Kolmogorov test (Supplemental Fig. S12), and subsequent consolidation into six macrostates via PCCA+ (Supplemental Fig. S13), transitions observed within the clustered trajectories were mapped to a discrete-state network, providing a kinetic model of the system's conformational dynamics (Fig. 11). While the underlying MD data supply the structural basis, the MSM partitions this conformational landscape into six kinetically distinct macrostates (MSM CS1–CS6) that clarify which transitions are facile and which represent kinetic traps.

Consistent with previous interpretation of the estimated PC1/PC2 free energy profile, a striking feature of the model is MSM CS1 as a kinetically isolated state. CS1 serves as a deep local basin stabilized by a persistent hydrogen-bonding scaffold in which the A10 phosphodiester anchors C8, U9, and C11. This network locks the loop in a distinct closed configuration. Occasional transitions from more flexible substates (MSM CS6 and CS4) funnel structures into CS1, but once populated, this substate is seldom vacated, underscoring its role as a kinetic sink within the loop landscape.

In contrast, most loop rearrangements unfold between and within the more flexible states corresponding to States A, B, and C. Within State B/C, MSM CS6 (43%) and MSM CS4 (16%) dominate the conformational sampling, which interconvert through modest reorganization of base stacking and loop opening, consistent with transient partial melting that preserves the overall folded topology. Equilibration within this B/C basin is frequent and rapid relative to the rare excursions into CS1.

Transitions between the State B/C substates and State A, defined by MSM CS2 (2.4%) and CS3 (7.6%), rely on more substantial loop rearrangement, revealing a loose but evident dependency on the specific stacking geometry to bridge these topologies. Within State A, CS2 and CS3 interconvert readily through local stacking shifts, but interestingly, CS3 never samples backward transitions into MSM CS4, implying that once the loop locks into the CS3 topology, its conformational barrier for that form of reversal is too high to overcome on the sampled MD timescale. Instead, transitions between the states predominantly proceed via CS2, which connects both to CS3 and back to the B/C network. Prior population refinement shows CS5 contributes little to the true solution-phase ensemble once ribose-sensitive ^3^J constraints are imposed, suggesting its role may be mainly kinetic rather than thermodynamic.

Taken together, this kinetic map (Fig. 11) demonstrates that loop dynamics cannot be captured by secondary structure populations alone. Frequent interconversion within each state preserves local stacking heterogeneity, while the more constrained pathways bridging State B/C and State A depend on collective loop rearrangements. The deep kinetic isolation of CS1 reinforces that certain substates, once formed, remain trapped unless large-scale backbone or hydrogen bond unwinding occurs, which are too slow on the timescale of our MD. Ultimately, the MSM highlights that dynamic equilibrium within and between substates reflects an interplay of local stacking motifs and global loop topology, which together define the accessible folding landscape.

### Conclusion

The intrinsic dynamics of most RNAs are difficult or impossible to capture by single structures or smaller structure bundles. RNAs in general can best be described by ensembles with interconverting conformations (Vicens and Kieft 2022). It was shown before that even a model hairpin with a thermodynamically stable tetraloop cannot be described accurately by a single structure (Oxenfarth et al. 2023). Our results regarding s2m_omicron agree with this notion. Despite consisting of a short stem with an apical pentaloop, the relatively small s2m_omircon RNA expresses significant dynamics. These dynamics are evidenced by high hetNOE values and conformational averaging of NMR parameters including NOEs, CCRs, and ^3^J-coupling constants. We investigated the ribose conformation of the loop nucleotides in detail by analyzing the chemical shifts in the form of canonical coordinates and by measuring ribose ^3^J-coupling constants (H1′H2′ and H3′H4′). Our results show that the loop opening nucleotides C8 and A12 behave similarly to the stem nucleotides as shown by a lower hetNOE value and stem-like canonical coordinates especially in the case of C8. For both nucleotides, we determined C3′-endo conformation to be the major populated conformation (C8: ∼95%, A12: ∼80%) that is typical for A-form helical regions. As expected, conformational averaging is more pronounced for the rest of the loop (U9, A10, C11), which is reflected in averaged ^3^J-coupling constants and canonical coordinates between expected values for C2′- and C3′-endo. While J-coupling constants are averaged over several milliseconds, ribose repuckering takes place on a subnanosecond timescale (Duchardt et al. 2008).

We were able to determine the main conformations for each nucleotide and calculated an NOE-based NMR solution structure. We included various dihedral restraints that were derived from different CCRs and J-coupling constants. The resulting structure bundle consists of three main states that are characterized by the relative position of A10, C11, and A12. However, these structures cannot represent the whole conformational space and need to be interpreted as the main conformations of the RNA. To expand our knowledge of the full ensemble of structures, we carried out MD simulations with the NMR structure bundle as a starting point. Close inspection of the MD-ensemble via principal component analysis and clustering revealed a large variety of loop conformations with the most prominent features being a A10–A12 stacking interaction and a A12/C8 Hoogsteen/sugar-edge base pair. Construction of a Markov state model revealed various transitions between the different conformational substates highlighting the dynamic nature of the pentaloop. The MSM ultimately strengthens our interpretation of the roles of the NMR states A, B, and C within the solution ensemble. NMR state A most closely corresponds to MD CS5, which is a dynamic and metastable CS that mediates transitions between other CSs stabilized by the presence of A10/A12 nucleobase stacking and A12-C8 noncanonical base-pairing, as in NMR state B or state C. The presence of secondary structure dynamics within each CS, particularly changes in A10 base stacking, is reflective of the differences noted between NMR states B and C. In this manner, our observed NMR states are structurally expanded through a diverse collection of MD-derived substates, facilitating experimental interpretation.

Consistent with the NMR data, analysis of the glycosidic torsion angle and back-calculated dipole(H1′-C1′)-dipole(H6/8-C6/8)-CCRs allowed us to explain the relatively high CCR of U9 by a mixture of syn- and anti-conformations. Similarly, a mix of conformations can also be observed for A10 and C11 although the syn-conformation is significantly less populated. To further refine the MD ensemble and improve agreement with the experimental ribose ^3^J-coupling constants, we used Bayesian/maximum entropy (BME) reweighting as done before for a UUCG tetraloop (Oxenfarth et al. 2023). Especially for the loop nucleotides, this improved agreement with the experimentally determined rates and C2′- and C3′-endo populations. Sugar pucker heterogeneity is preserved for U9 and C11 where also the NMR data (^3^J-coupling constants and canonical coordinates) suggest fast averaging between C2′- and C3′-endo. After BME reweighting, for C8 and A12, relatively clear C3′-endo conformations and a C2′-endo main conformation for A10 are strengthened in the MD-ensemble proving the effectiveness of the approach.

Although the secondary structure of s2m_omicron differs significantly from the ones of s2m_delta and s2m_Wuhan, one feature is conserved. The tip of both loops consists of the same three nucleotides U, A, and C, and for all s2m variants, significant dynamics are present for these nucleotides (Matzel et al. 2024; Martin et al. 2025). Possibly, this dynamic UAC base triplet is crucial for the still unknown function of s2m. Given that structural ensembles of RNAs in general are essential to facilitate the underlying features for the identification and understanding of biological function (Al-Hashimi and Walter 2008; Cruz and Westhof 2009; Ganser et al. 2019), our work contributes to the pool of knowledge necessary to make novel structural and dynamics connections to reveal biological function of s2m.

## MATERIALS AND METHODS

### RNA sample preparation

The s2m_omicron DNA plasmid was prepared via cloning of hybridized complementary oligonucleotides into the pSP64 vector containing the HDV ribozyme sequence. The restriction sites *EcoRI* and *NcoI* were used for this. The plasmid was kindly provided by Professor Dr. Julia Weigand as part of the Covid-19 NMR consortium. Standard protocols for plasmid amplification were applied as described before (Schnieders et al. 2020). The RNA samples were prepared via in vitro transcription using an in-house-produced T7 polymerase. The RNA was purified using preparative polyacrylamide gel electrophoresis (PAGE) with subsequent *rp*-HPLC and rebuffering in the NMR buffer. Before measuring, a folding protocol was carried out including heating to 95°C for 5 min and cooling down to room temperature. The sample homogeneity and monomeric state of the RNA were ensured with denaturing and native PAGE. A list of all samples is given in Supplemental Table S2. A detailed description of the RNA preparation including all needed protocols was described before (Schnieders et al. 2020).

### NMR measurements

All spectra were recorded with samples containing 25 mM K_2_HPO_4_/KH_2_PO_4_ (pH 6.2) and 50 mM KCl at various temperatures between 278 and 298 K. 2,2-Dimethyl-2-silapentane-5-sulfonate (DSS) was used as an external reference. All samples were measured in 300 µL in 5 mm Shigemi NMR tubes. Measurements were carried out at different Bruker spectrometers equipped with 5 mm cryo probes: two 600 MHz spectrometers (Avance III HD, Prodigy TCI ^1^H/^19^F,^15^n,^13^C and Avance Neo, TCI ^1^H,^15^n,^13^C), two different 800 MHz spectrometers (Avance III HD, TCI ^1^H,^15^n,^13^C and Avance III, TXO cryo ^13^C,^1^H,^15^n), and a 700 MHz spectrometer (Avance III HD, QCI cryo ^1^H,^15^n,^13^C,^31^P). Detailed experimental parameters for all experiments carried out are given in Supplemental Table S2. Processing and analysis of the spectra was done with TopSpin 4.1.4, and NMRFAM-SPARKY (Lee et al. 2015) was used for the resonance assignment. The assignment is deposited in the BMRB (ID: 34991).

### Heteronuclear NOE measurements

All hetNOE data were recorded with sample #3 (Supplemental Table S1) at 298 K on a 600 MHz spectrometer. The ^1^H, ^13^C HSQC-based hetNOE spectra were recorded as pseudo 3Ds with the NOE and reference spectra recorded in an interleaved manner. The relaxation delay was set to 5 sec, a presaturation delay of 3 sec was applied, and an off resonant pulse at −4000 ppm was used in the reference experiment for temperature compensation. Separate experiments were carried out for the aromatic H6/8–C6/8 and the ribose H1′-C1′ resonances. The hetNOE was calculated based on the intensities of the spectra as the ratio of the intensities of the NOE spectrum to the noNOE spectrum. The errors (Δ_hetNOE_) were calculated based on the signal-to-noise ratios with the following formula:

$$\Delta_{\mathrm{hetNOE}}=\sqrt{{\left(\frac{I_{\mathrm{NOE}}}{I_{\mathrm{ref}}^{2}}\times N_{\mathrm{ref}}\right)}^{2}+{\left(\frac{1}{I_{\mathrm{ref}}}\times N_{\mathrm{NOE}}\right)}^{2}}$$

*I*_NOE_, intensity of the NOE spectrum; *I*_ref_, intensity of the reference spectrum; *N*_NOE_, noise of the NOE spectrum; *N*_ref_, noise of the reference spectrum.

### Calculation of chemical shift perturbations

For the quantification of temperature-induced chemical shift changes from 278 to 298 K, the Euclidean distance (*d*) was calculated based on the following formula:

$$d=\sqrt{\frac{1}{2}(\delta_{H}^{2}+\alpha \times \delta_{C}^{2})}$$

*d*, Euclidean distance; δ_H_, chemical shift changes of the ^1^H chemical shifts; δ_C_, chemical shift changes of the ^13^C chemical shifts; α, weighting factor based on the ratio of the gyromagnetic ratios α = 0.25 as described previously (Getz et al. 2007).

### Canonical coordinates

Based on the NMR assignment of the ^13^C resonances of the ribose moieties, canonical coordinates (can1* and can2*) were calculated as published before (Ebrahimi et al. 2001; Cherepanov et al. 2010) with the following formulas:

$$\begin{array}{rl} \mathrm{can}1^{*} & =P_{\mathrm{Fit}}=-14.7\delta_{C1^{\prime }}+22.1\delta_{C2^{\prime }}+13.2\delta_{C3^{\prime }}+6.5\delta_{C4^{\prime }}\\ & \;-2.9\delta_{C5^{\prime }}-1595 \end{array}$$

$$\begin{array}{rl} \mathrm{can}2^{*} & =\gamma_{\mathrm{Fit}}=9.8\delta_{C1^{\prime }}+16.5\delta_{C2^{\prime }}-0.5\delta_{C3^{\prime }}-1.7\delta_{C4^{\prime }}\\ & +13.5\delta_{C5^{\prime }}-2781 \end{array}$$

P_Fit_, predicted pseudorotation phase; γ_Fit_, predicted torsion angle; δ_C1′-C5′_, ^13^C chemical shift in ppm.

### Determination of the population of loop nucleotides in C3′-endo based on ^3^J-coupling constants

To estimate the percentage of C3′ endo conformation for the loop nucleotides, we globally fitted the averaged experimental couplings to a two-state model. Values for the two states pseudorotation phases of 18° and 162° (Altona and Sundaralingam 1972) and the corresponding ^3^J-coupling constants (Haasnoot et al. 1980; Marino et al. 1999) were used as values for C3′- and C2′-endo conformations. Averaged rates were calculated for different percentages of C3′-endo conformation from 1% to 100% and the experimentally determined couplings were subtracted. The sum of all differences was ultimately plotted against the population, and the minima of the curves was interpreted as the percentage of nucleotides that adopt a C3′-endo conformation (Fig. 4C). In this two-state model, the rest of the structures are considered to adopt a C2′-endo conformation. To investigate how the exact values of *P* for C2′- and C3′-endo influence this fit, all possible combinations of *P* = 8°, 18°, 28° and *P* = 152°, 162°, 172° were tested (Supplemental Fig. S3).

### NMR structure calculations

The structure calculations of s2m_omicron were performed with ARIA 1.2 (custom web portal based on Linge et al. 2003) with the implementation of NOEs and dihedral restraints. The NOEs were derived from five different 2D NOESY spectra, two of them measured on H_2_O samples with mixing times of 100 and 150 msec and three of them measured on D_2_O samples with mixing times of 50, 100, and 150 msec. For the dihedral restraints (α, β, γ, δ, ε, ζ, χ, and the pseudorotation phase P in the form of ν_0_–ν_4_), standard canonical A-form-helix values were used for the stem nucleotides. For the loop nucleotides, β, γ, ε, χ, and P were determined experimentally, as described in the Results section. A list of all dihedral restraints applied is given in Supplemental Tables S5 and S6. In general, ranges of angles were used as restraints rather than exact angles. In addition, H-bond and planarity restraints were used for the stem nucleotides, where we confirmed Watson–Crick base-pairing with NOESY and HNN-COSY spectra, as described in the Results section. A final number of 20 iteratively refined structures was calculated, while in the process, 100 structures were calculated in each iteration and 200 structures in the last cycle. Standard protocols and settings optimized for RNA were used. Optimized potentials for liquid simulation (OPLS) (Jorgensen et al. 1996) were used for water refinement of the final bundle containing the 20 lowest energy structures. HydroNMR (Garcı´a de la Torre et al. 2000) was used to estimate the hydrodynamic radius of the RNA. The final structure bundle was clustered visually into three substates based on the relative configuration of the loop nucleobases A10 and C11. The pairwise mean RMSD was calculated with MOLMOL (Koradi et al. 1996). The complete structure bundle is accessible via the PDB (code: 9QZJ).

### Molecular dynamics simulations

Simulations were carried out with the NAMD 2.14 engine, using the AMBER nucleic acid force field with parameterization derived from AMBER99, PARMBSC0, and χOL3 (Cornell et al. 1995; Cheatham et al. 1999; Phillips et al. 2005, 2020; Pérez et al. 2007; Zgarbová et al. 2011). Ten simulations were carried out, each from a distinct ARIA structure, ensuring representation from NMR states A (three structures), B (one structure), C (two structures), and one unique structure from each of the three “exotic states” (Rieping et al. 2007). To address known AMBER parameterization flaws for 5′-terminal monophosphate capping, a terminal phosphate oxygen was deprotonated, resulting in a net charge of −20 for the simulated RNA (Bruist and Case 2023). Consequentially, our s2m systems were charge neutralized through the addition of 20 K^+^ modeled by Li/Merz ion parameters and solvated with 15 Å padding of TIP3P water (Jorgensen et al. 1983; Li et al. 2013, 2015a,b; Li and Merz 2014). Each system was simulated under the NPT ensemble at 310 K and 1 atm, using the Nosé–Hoover Langevin piston barostat (Martyna et al. 1994; Feller et al. 1995). Each system was subject to 1000 steps of conjugate gradient energy minimization and equilibrated for 50 nsec. Subsequently, each of the 10 production simulations was run for 1 µsec, for a total of 10 µsec of simulation data. Coordinates were saved every 200 psec, yielding 50,000 simulation frames for analysis.

### Principal component analysis

Concatenated simulated data from the 10 simulations were used to compute covariances for principal component analysis (PCA), wherein highly dimensional data are projected to a much lower dimensional linear subspace capturing the largest fraction of variance in the data, as indicated by a scree plot (Manly and Navarro Alberto 2016; Brunton and Kutz 2019). This procedure ultimately yields a depiction of molecular structural information separated by relative similarity and dissimilarity (Stein et al. 2006). We performed PCA on the nonhydrogen atoms within the terminal pentaloop (nt 8–12). Approximately 50% of Cartesian coordinate variance was captured within two or three principal components for any index of selectivity. Following dimensionality reduction with PCA, a Gaussian mixture model (GMM) was used to estimate the density of sampling in principal component (PC) space (Dempster et al. 1977; McLachlan and Peel 2000). The GMM density estimate was further used to approximate the relative free energy associated with the marginal PC1–PC2 configuration space (Altis et al. 2008; Papaleo et al. 2009). Spectral or *k*-means clustering was applied to partition simulation data into conformational substates (CSs), where the clustering method and the number of clusters were selected to align as best as possible with the basins identified in the free energy landscape (MacQueen 1967; Lloyd 1982; Hamad and Biela 2008). The centroid of each CS was computed as a representative for structural analysis, and dynamic fluctuations within each CS were quantified by calculating the average root mean squared fluctuation (RMSF) per nucleotide relative to the CS centroid. This was facilitated by an in-house Python script, using the libraries of MDTraj to parse simulation data; Scikit-learn to perform PCA, GMM optimization, and clustering; and Matplotlib for graphics (Hunter 2007; Van Rossum and Drake 2009; Pedregosa et al. 2011; McGibbon et al. 2015). Molecular visualization was done with VMD (Humphrey et al. 1996). Dynamic secondary structures for each CS were measured using the Barnaba Python library (Bottaro et al. 2019).

### Markov state model

A Markov state model (MSM) was constructed from pentaloop dihedrals from our MD trajectories using the deeptime Python library (Hoffmann et al. 2022). To reduce the dimensionality of the dihedral angle space, time-independent component analysis (TICA) was used to extract kinetically distinct states (Molgedey and Schuster 1994; Naritomi and Fuchigami 2013). The reduced dimensionality space was subsequently clustered using the *k*-means algorithm, with initial centroids selected by the *k*-means++ scheme to improve convergence and cluster quality, where the number of microstates was set to *k* = 300 (Arthur and Vassilvitskii 2007). To validate the choice of lag time, implied timescales were computed, demonstrating convergence at a lag time of ∼30 nsec (Bowman et al. 2009). Consistency of the MSM kinetics was confirmed by performing a Chapman–Kolmogorov test (Prinz et al. 2011), verifying that the model accurately reproduces transition probabilities at multiples of the chosen lag time (see Supplemental Fig. S12).

The discrete microstates were coarse-grained into six kinetically metastable macrostates using Perron Cluster Cluster Analysis (PCCA+) (Röblitz and Weber 2013), which identifies optimal partitions of the state space based on the leading eigenvectors of the transition probability matrix (Supplemental Fig. S13). To further characterize the structural features of each macrostate, the Barnaba Python library was used to extract representative secondary structures (Bottaro et al. 2019), providing structural context to the kinetic model. Finally, the coarse-grained transition probability matrix for the six-state MSM was computed, enabling characterization of the equilibrium populations and the dominant transition pathways between macrostates, thereby revealing the interdependence of the principal conformational states of the terminal pentaloop.

### Bayesian maximum entropy reweighting

The Bayesian/maximum entropy (BME) reweighting method was used to generate a reweighted ensemble in better agreement with pentaloop ^3^J-coupling constants by assigning each structure from MD an updated statistical weighting (Bottaro et al. 2020; Bernetti and Bussi 2023). The BME objective function balances minimizing the χ^2^ deviation between experimental ^3^J-coupling constants and simulated averages with an entropy term, scaled by a hyperparameter θ, that penalizes overfitting to a small collection of structures, preserving ensemble diversity. ^3^J-coupling constants were derived using an in-house Python script (Richter et al. 1999). BME optimization was performed using the BME Python library, and the entropy scaling hyperparameter was set to 3 to greatly improve pentaloop ^3^J-coupling constants while maintaining a relatively large proportion of effective frames from the original MD data (∼30%) (Bottaro et al. 2020). Structural implications of reweighting were studied by sampling 50,000 frames with replacement using weights from the BME results, implemented with NumPy and MDTraj (McGibbon et al. 2015; Harris et al. 2020).

## DATA DEPOSITION

NMR raw data and MD centroid PDB files are available at the Goethe University Frankfurt Data Repository GUDe. The NMR structure bundle is available at the PDB (code: 9QZJ), and the chemical shift assignment is available at the BMRB (ID: 34991).

## SUPPLEMENTAL MATERIAL

Supplemental material is available for this article.

## Figures and Tables

**Tobias Matzel:**
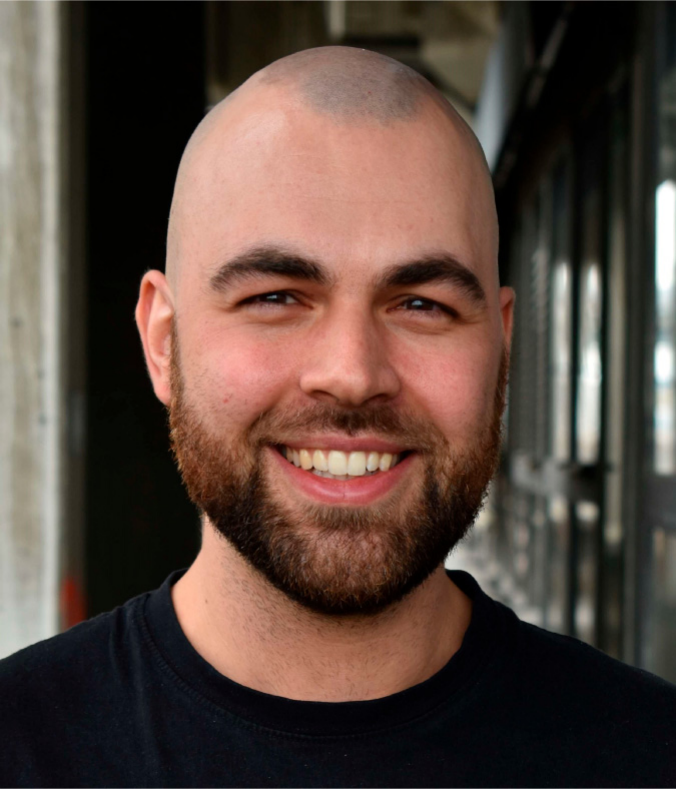


**Joseph A. Makowski:**
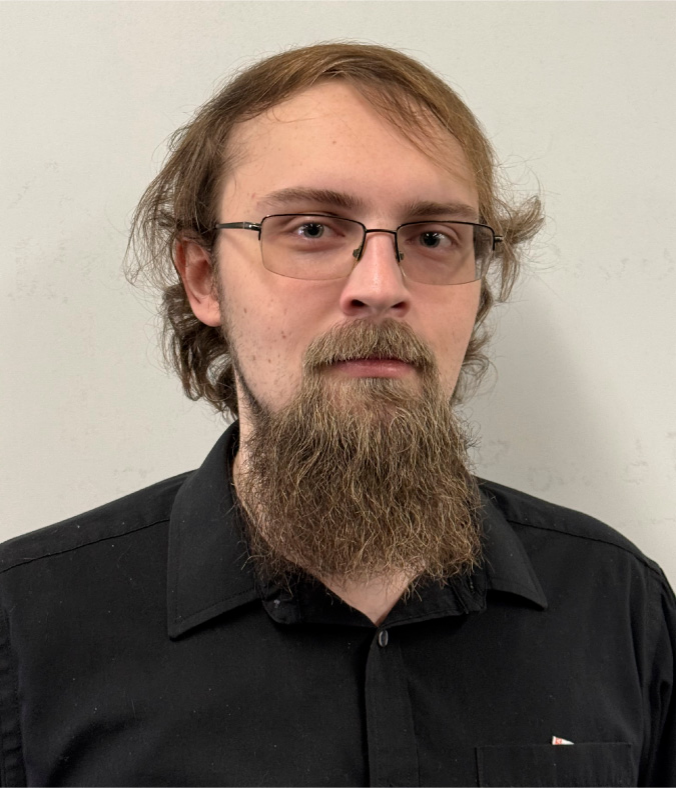


## MEET THE FIRST AUTHORS

***Meet the First Author(s)* is an editorial feature within *RNA*, in which the first author(s) of research-based papers in each issue have the opportunity to introduce themselves and their work to readers of *RNA* and the RNA research community. Tobias Matzel and Joseph A. Makowski are co-first authors of this paper, “Integrated NMR and MD structure and dynamics of the stem–loop-II motif (s2m) from the Omicron variant of SARS-CoV-2.” Tobias is a former PhD student in the Department of Organic Chemistry and Chemical Biology at Goethe University Frankfurt. Within the Schwalbe group, his focus was using NMR spectroscopy to gain information about the structure, dynamics, and interactions of RNAs with other biomolecules or small molecules. Joseph is a PhD student in the Department of Chemistry and Biochemistry at Duquesne University, where he studies the structure and dynamics of RNA. Joseph's work combines molecular dynamics simulations with experimental data using contemporary quantitative approaches to gain a deeper understanding of how RNA elements may function through a diverse ensemble of structures.**

**What are the major results described in your paper, and how do they impact this branch of the field?**

We determined the first NMR solution structure of the Omicron stem–loop-II motif (s2m) and, importantly, complemented it with molecular dynamics simulations refined by experimental data. This combined approach showed that, even though the Omicron s2m is truncated compared to earlier variants, it retains substantial conformational dynamics. In particular, we observed multiple stacking patterns, ribose repuckering, and reorganization of a conserved UAC triplet at the loop tip. By integrating structural and dynamical views, our work highlights how viral RNA elements can maintain functional plasticity even after major sequence and structural alterations. This type of insight is crucial for understanding not only the behavior of s2m but also, more broadly, how RNA viruses preserve critical regulatory features while undergoing rapid evolutionary change.

**What led you to study RNA or this aspect of RNA science?**

**TM:** During my master's studies in Frankfurt, I focused on NMR, as this has always interested me. Fortunately, Frankfurt offers access to several high-field NMR spectrometers and a lot of expertise in this field. Beginning with my master's thesis in the Schwalbe group, I investigated RNA–protein interactions with the protein side as the readout. Although I enjoyed my initial projects, I recognized the need to expand my knowledge of NMR on RNA. This method is particularly effective in this field as RNAs often contain highly dynamic regions that can only be correctly described when structural investigations are conducted in solution.

**JAM:** I have always been drawn to problems that sit at the intersection of chemistry, physics, and mathematics. When I began my PhD at Duquesne, I was intrigued by the Evanseck Research Group's work on the s2m element because it combined these perspectives in a very tangible way. MD simulations coupled with appropriate statistical or quantitative methods are powerful tools for studying highly dynamic RNA and an area where one has freedom to approach research problems in diverse ways. For me, studying RNA felt like the natural place to bring together my background in computation and theory with the challenge of understanding biologically important molecules.

**During the course of these experiments, were there any surprising results or particular difficulties that altered your thinking and subsequent focus?**

**TM:** When we started this project, I thought of it as being relatively straightforward to determine the solution structure of a relatively small RNA with NMR. The resonance assignment was done quickly, and the quality of the spectra looked promising. It was planned as a 6 month project after the complete assignment. When we looked at the first structure calculations and the hetNOEs, we slowly began to realize that this might be a bigger project than initially thought. We started with further experiments to determine dihedral restraints and observed averaging of NMR parameters. At that point, we were certain that we somehow had to take the dynamics into account. Luckily, we were collaborating with the Evanseck lab on another project and knew what we had to do from there.

**What are some of the landmark moments that provoked your interest in science or your development as a scientist?**

**TM:** During my primary school years, I developed a keen interest in science documentaries. In the following years, people that influenced me the most were my biology, chemistry, and physics teachers. One incident that stands out was when my chemistry teacher moved me to the front row due to my disruptive behavior with my friends. Before that day, I was mainly interested in biology and was not really good at chemistry. But when I sat there alone with no disturbance, I started to really follow class. From that point on, I realized that chemistry was remarkably interesting, and my grades in chemistry, math and physics improved significantly. An internship at another group from Goethe University led to the decision to study chemistry with the possibility to specialize in biochemistry.

**If you were able to give one piece of advice to your younger self, what would that be?**

**JAM:** From the beginning, I placed a high value on having both breadth and depth across different fields, because I wanted to enjoy the benefits of approaching problems from multiple perspectives. That mindset came from a genuine curiosity, but it also meant I had a tendency to overzealously commit myself to too many things at once. Looking back, I would tell my younger self that it is better to focus more deliberately on the problems and directions that matter most, and to develop them properly, rather than spreading myself so thin that nothing could be pursued as well as it might have been.

**What are your subsequent near- or long-term career plans?**

**JAM:** I am interested in pursuing a career that allows me to continue working at the intersection of computation, theory, and molecular science. In the near term, I am exploring opportunities both in academia and industry where I can apply my skills in molecular simulation and data science to challenging problems. Longer term, I hope to develop a career that balances research, collaboration, and mentorship, and I remain open to a broad range of directions where I can make a meaningful contribution.

**What were the strongest aspects of your collaboration as co-first authors?**

The strongest part of our collaboration was how naturally our different areas of expertise fit together. While TM approached the problem from the NMR side and JAM from molecular dynamics, we maintained clear and structured communication throughout. Rather than working in parallel, we treated the experimental and computational results as pieces of the same puzzle, which allowed us to refine ideas iteratively and play to the strengths of each method. That complementarity made the project more rewarding and drove improved physical and chemical insight of the Omicron s2m.
